# Supplementary material for: Impact of early locus coeruleus lesions in the TgF344 Alzheimer's disease rat model
Source: Alzheimers Dement. 2026 Apr 6;22(4):e71334. doi: 10.1002/alz.71334 (PMC13053930; doi:10.1002/alz.71334)
Supplement: Supplementary file 2 — Supporting Information [file ALZ-22-e71334-s001.docx]

*Table 1. Antibody information*

| **Primary Antibody** | **Vendor**  **(Catalog #)** | **Dilution** | **Secondary Antibody** | **Vendor**  **(Catalog #)** | **Dilution** |
| --- | --- | --- | --- | --- | --- |
| Mouse anti-CP13 | Gifted from Peter Davies | 1:2000 | Goat anti-mouse 568 | ThermoFisher Scientific  (A-11004) | 1:500 |
| Mouse anti-4G8 | BioLegend  (800798) | 1:1000 | Goat anti-mouse 568 | ThermoFisher Scientific  (A-11004) | 1:500 |
| Rabbit anti-IBA1 | Fujifilm  (019-1974) | 1:1000 | Goat anti-rabbit 488 | ThermoFisher Scientific  (A11008) | 1:500 |
| Mouse anti-NET | Mab Technologies  (NET05-2) | 1:500 | Goat anti-mouse 568 | ThermoFisher Scientific  (A-11004) | 1:500 |
| Rabbit anti-GFAP | Abcam  (ab7260) | 1:1000 | Goat anti-rabbit 488 | ThermoFisher Scientific  (A11008) | 1:500 |
| Chicken anti-TH | Abcam  (ab76442) | 1:1000 | Goat anti-chicken 647 | ThermoFisher Scientific  (A-21449) | 1:500 |


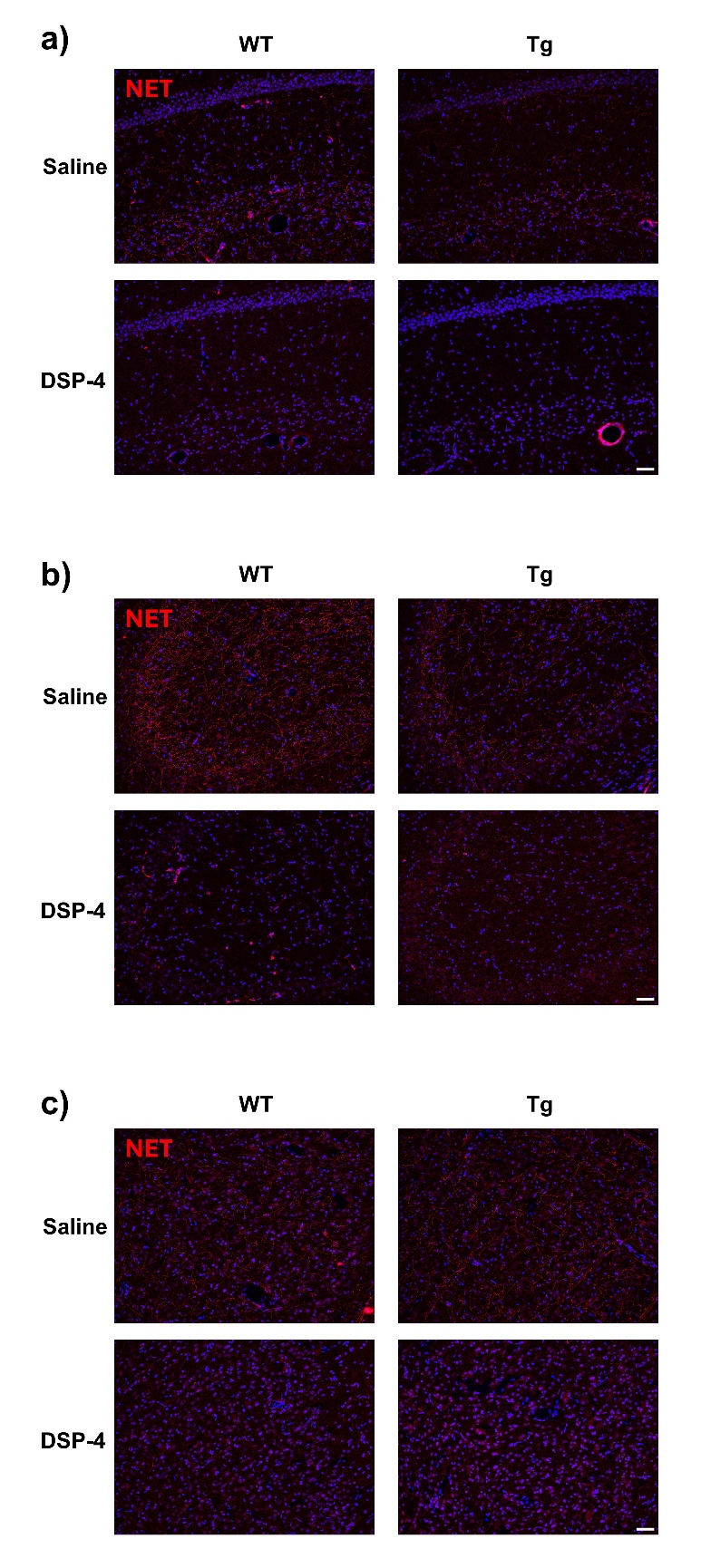


**Supplemental Figure 1. Representative images of NET staining in the forebrain.** Representative images (20x) of NET+ fibers in the CA1 (a), CA3 (b), and PFC (c) with a DAPI counterstain. Scale bar = 50 µm.


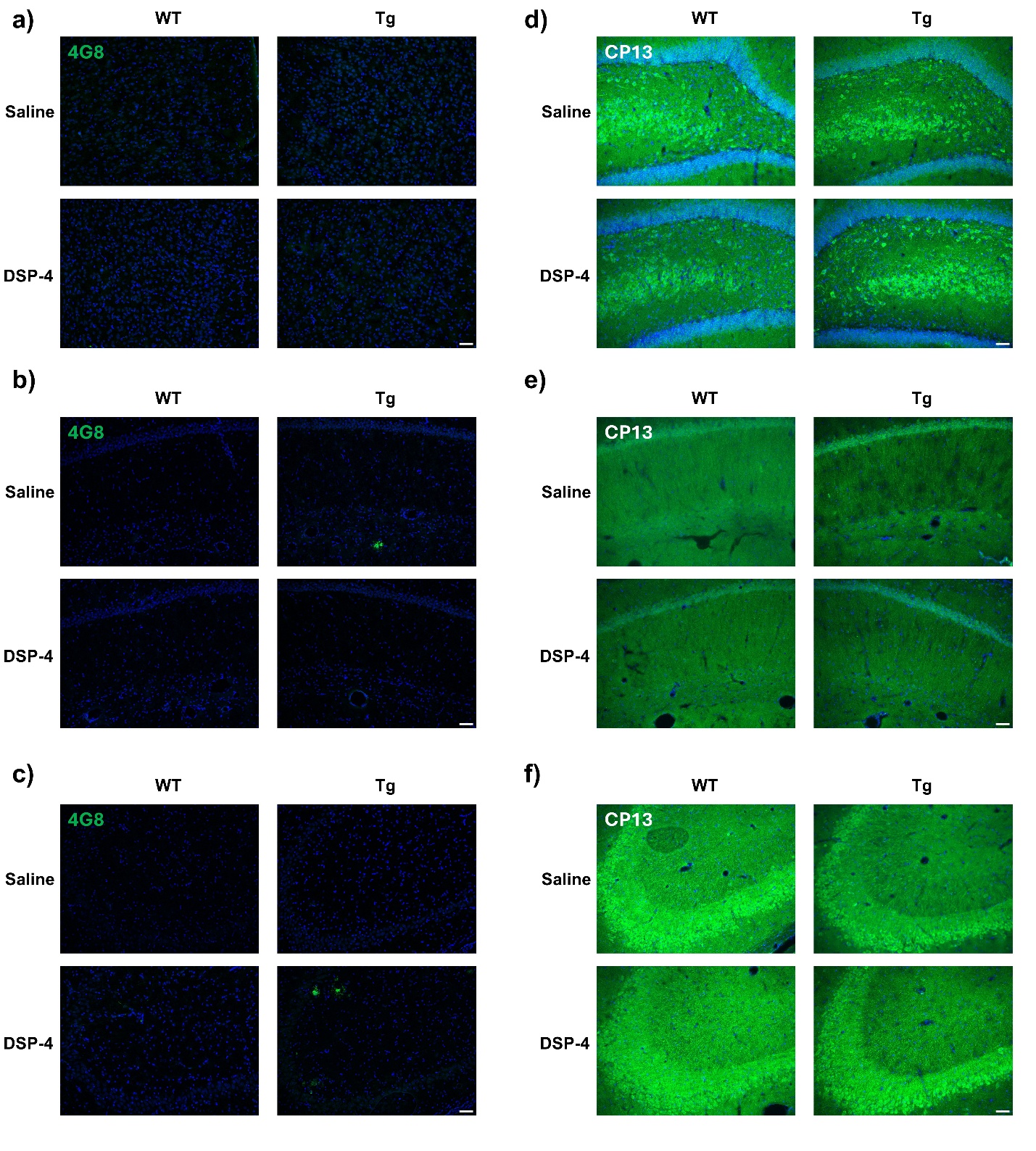


**Supplemental Figure 2. Representative images of AD-like neuropathology in the forebrain.** Representative images (20x) of amyloid-β pathology in the PFC (a), CA1 (b), and CA3 (c) with a DAPI counterstain. Representative images (20x) of CP13 staining in the DG (d), CA1 (e), and CA3 (f) with a DAPI counterstain. Scale bar = 50 µm.


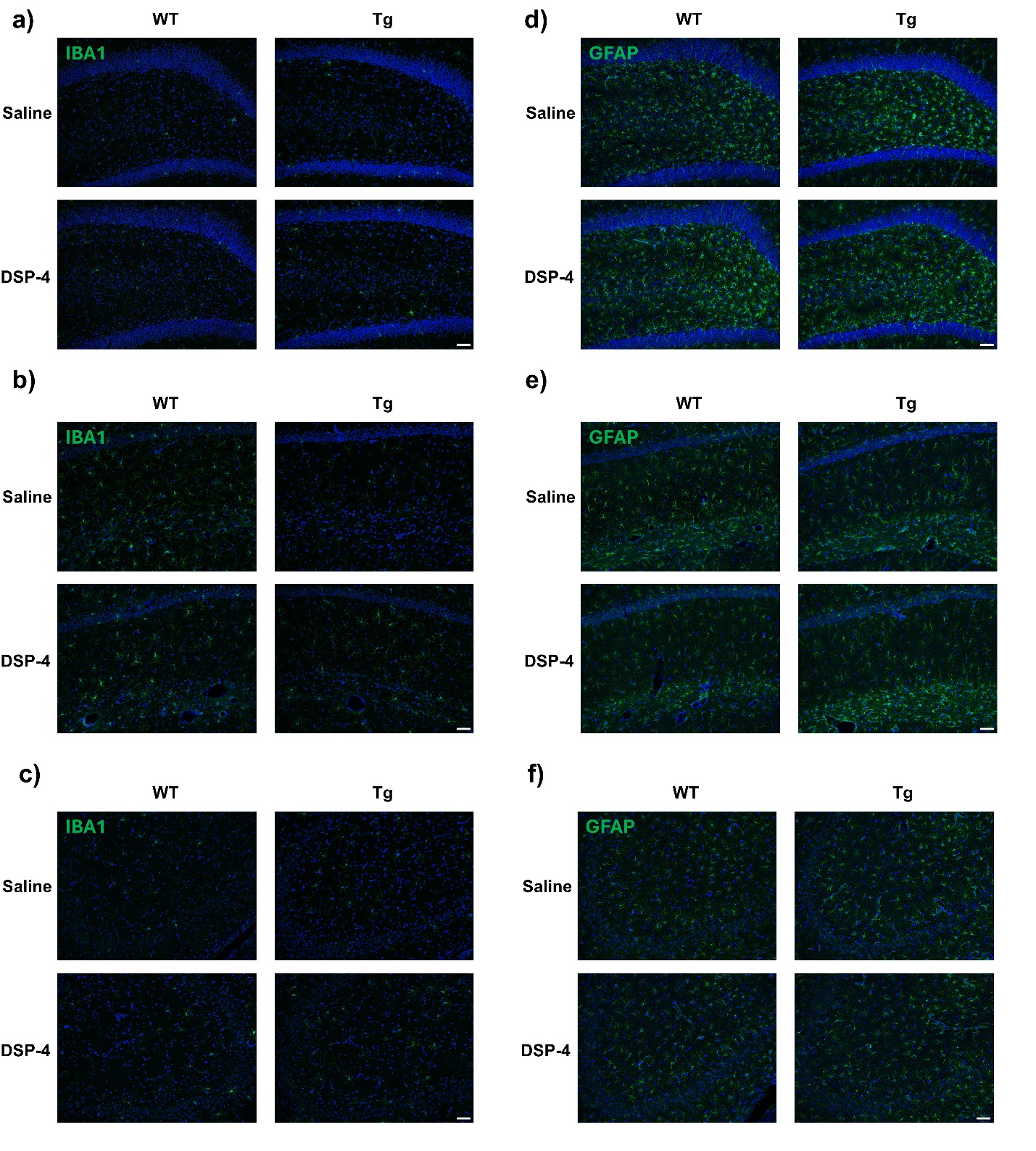


**Supplemental Figure 3. Representative images of astrocytic and microglial neuroinflammation in the forebrain.** Shown are representative images (20x) of IBA1 staining in the DG (a), CA1 (b), and CA3 (c) with a DAPI counterstain. Representative images (20x) of GFAP staining in the DG (d), CA1 (e), and CA3 (f) with a DAPI counterstain. Scale bar = 50 µm.
